# Flipping the script: Using mucosal immune obstacles to inform vaccine design

**DOI:** 10.1371/journal.ppat.1014230

**Published:** 2026-05-26

**Authors:** Avan Antia, Siyuan Ding

**Affiliations:** Department of Molecular Microbiology, Washington University School of Medicine, Saint Louis, Missouri, United States of America; University of Iowa, UNITED STATES OF AMERICA

The lag in oral vaccine development is often attributed to platform limitations, as subunit or non-replicating oral vaccines typically elicit weak responses in the gut. This helps explain why currently licensed mucosal vaccines are exclusively live-attenuated. However, many barriers to effective oral vaccine design stem from the fundamental properties of the mucosal immune system itself. The gut has evolved to prioritize barrier integrity and tolerance over the production of sterilizing immunity, creating an inherent mismatch between the architecture of gut-associated lymphoid tissue (GALT) and traditional vaccination goals. Defining the constraints of the mucosal immune landscape and understanding how pathogens naturally overcome them offer an opportunity to rethink oral vaccine development through the lens of mucosal immunology rather than vaccine platforms alone.

## *Understanding the system*: What are the challenges of the mucosal immune system that hinder successful oral vaccine development?

Unlike the systemic immune system, which operates in relatively sterile compartments, the gut continually encounters environmental antigens, dietary components, commensal microbes, and potential pathogens. This persistent pressure has driven the evolution of the gut’s primary immune response to be biased towards tolerance. Oral tolerance, as originally described over a century ago, refers to the systemic hypo-responsiveness induced by antigens that are initially introduced orally. Today, the more generally defined **mucosal tolerance** has expanded to encompass our growing understanding of the coordination between the GALT and its cellular and chemical components, resulting in homeostatic hypo-responsiveness. Mechanistically, mucosal tolerance is mediated by several mechanisms, including the induction of regulatory T cells, T cell anergy, the production of immunoregulatory cytokines such as IL-10, and the tolerogenic programming of intestinal dendritic cells.

Superimposed upon tolerance, preexisting **immune tone** also biases mucosal responses. Immune tone can be shaped by prior pathogenic exposure, as well as microbial makeup. For example, a commensal murine astrovirus can stimulate host anti-viral interferon-λ responses and protect immunodeficient mice from rotavirus and norovirus infection [1], illustrating how infection history can affect subsequent responses to new pathogens. In humans, co-administration of oral polio vaccine with oral rotavirus vaccine results in decreased anti-rotavirus seroconversion [2]. Moreover, certain constituents of the enteric virome are associated with lower levels of rotavirus vaccine shedding [3], further demonstrating how immune tone can be affected by concomitant viral insults. Commensal bacterial elements can also shape immune tone. Weaning, or the transition to solid foods around 6 months of age, represents the largest microbial shift in life, after which an “adult-like” microbiome develops over the first 5 years [4]. Recently, it has been demonstrated how this weaning-mediated microbial shift epigenetically reprograms MHC-II expression in intestinal stem cells, highlighting the lasting effects of the microbiome on immune memory [5]. Importantly, weaning-mediated changes in immune tone may influence responses to oral vaccines administered in early life. Further, segmented filamentous bacteria can modulate immune responses to protect against rotavirus infection through increasing epithelial cell turnover [6] and against respiratory virus infections, including influenza virus, respiratory syncytial virus, and SARS-CoV-2, via effects on alveolar macrophages [7]. Finally, microbiota-derived metabolites, such as short-chain fatty acids, also modulate a variety of innate and adaptive lymphoid cells, once again setting immune tone for future responses [8].

An additional layer of complexity arises from the **route of priming** itself, as mucosal immunization is usually required to elicit mucosal response. All currently licensed rotavirus vaccines are oral drops containing live-attenuated viruses. By contrast, parenteral immunization strategies have yielded inconsistent results. Intramuscular immunization of mice with live or inactivated rotavirus particles induces robust serum IgG responses, but not intestinal IgA responses, and does not protect mice from challenge [9]. Epidermal immunization with microneedle patches coated with inactivated rotavirus triggers activation of memory B and effector memory T helper cells, and induces expression of one gut-homing receptor on these cells in the systemic compartment, however, mucosal responses were not investigated [10].

Importantly, mucosal responses are shaped by local imprinting cues, which restrict regions of **lymphocyte homing** after mucosal immunization. For example, oral introduction of antigen leads to imprinting of intestinal homing markers (*i.e.*, CCR9 and α4β7) onto activated lymphocytes, whereas intramuscular injection leads to systemic homing patterns, favoring trafficking to peripheral sites. There are certain known cellular homing networks connecting locations distinct from where priming occurs. GPR25 mediates lymphocyte homing to the CNS, respiratory, upper gastrointestinal, biliary, and genitourinary tracts [11], establishing one potential network between non-intestinal mucosal sites. Furthermore, CCR10 has been described as a “general” mucosal homing receptor, expressed on most IgA-secreting cells, allowing homing to diverse mucosae, including colon and mammary glands [12]. While various chemokine-receptor pairs have been identified (*i.e.*, α4β7-MAdCAM-1, CXCR4-CXCL12, and CCR7-CCL21), many unknowns remain in terms of regional expression and sharing between different sites. Investigation into homing receptors that are shared by multiple mucosae can help drive broadly protective vaccine design.

Together, these observations underpin the concept of an “**integrated mucosal immune system**,” in which immune responses at one mucosal site can influence immunity at other mucosal surfaces. Importantly, this connection is directional and context-dependent, rather than global. For example, circulating tonsillar plasmablasts possess a non-intestinal homing receptor profile, thus excluding these cells from the gut [13]. Thus, while mucosal sites are supposedly “connected,” immune cell trafficking between them is constrained by the nature of the antigen, local inflammatory cues, and the site of priming.

Collectively, these features illustrate why eliciting robust immune activation via oral vaccination is complex and challenging. Therefore, it is imperative to obtain a deep understanding of the system itself and deliberately incorporate its regulatory logic to inform vaccine development (**Table 1**).

**Table 1:** Considerations and alternatives for conventional vaccine platforms, specifically in the context of oral vaccine design.

In the context of an oral vaccine…			
Platforms	Pros	Cons	Alternatives & Considerations
***Subunit and inactivated vaccines***	• No potential for pathogenic reversion.	• Often not immunogenic enough to cause immune response when administered orally (*mucosal tolerance*).	• Add well-characterized mucosal adjuvants to break tolerance barrier [16–20]. • Prime parenterally and “pull” to modulate mucosal effects [29].
***Live-attenuated vaccine***	• Mimics natural infection and overcomes tolerance barrier.	• Potential for pathogenic reversion and vaccine-mediated disease. • Efficacy may vary based on mucosal infection and vaccination history and microbiome (*immune tone*).	• Direct M cell targeting may improve vaccine efficacy and decrease effective dose required [35]. • Addition of host-modulating components (*i.e*., small molecule inhibitors [36], probiotics [37,38], and prebiotics [37]) may improve efficacy.
***Replication-deficient viral vector***	• No potential for pathogenic reversion.	• May require high doses to elicit effective immune response in gut. • Efficacy may vary based on mucosal infection and vaccination history and microbiome (*immune tone*).	• Adjuvants and mucosal homing modulators can be encoded directly into vector [15,28]. • Consider using replicating enteric virus as vector (*i.e*., rotavirus [14]) to enhance immune response.
***mRNA***	• No potential for pathogenic reversion. • Shorter development and production time.	• Requires delivery systems that withstand low pH of stomach (*route of priming*).	• Prime parenterally and “pull” to modulate mucosal effects [29]. • Encode homing modulators (*i.e*., retinoic acid agonists [28]) directly into mRNA.

## *Hacking the system*: How can we use these challenges to inform smarter vaccine design?

### Overcoming tolerance

Viral vectors have the potential to enable multivalent protection by utilizing one virus to encode immunogens of other pathogens. In principle, this approach may overcome the tolerance threshold, as the vector itself mimics natural infection and contains pathogen-associated molecular patterns that act as natural adjuvants. Kawagishi and colleagues used reverse genetics systems to rescue recombinant rotaviruses encoding the human norovirus VP1 protein or P domain, and found that immunized mice elicit both systemic and mucosal neutralizing antibody responses [14]. This provides proof of principle to modify currently existing viral vaccines to include an immunogen from another enteric pathogen for which a vaccine does not yet exist. Similarly, adenovirus-vectored vaccines provide the benefit of avoiding potential pathogenic reversion as in the case of live-attenuated vaccines. An oral vaccine tablet containing a non-replicating recombinant human adenovirus-5 vector encoding the human norovirus VP1 antigen and a molecular double-stranded RNA adjuvant has been deemed safe and immunogenic in human clinical trials [15]. This vaccine induces antibody responses in the saliva and nasal washes, implying immune response at various mucosal sites, although stool responses were not measured. Finally, mucosal adjuvants can be used to surpass tolerance. While few are currently approved, there are various avenues worth investigating to find safe and effective mucosal adjuvants, such as U-Omp19 bacterial protease inhibitor [16–19], a double mutant heat labile toxin encoded by enterotoxigenic Escherichia coli, oral saponin, toll-like receptor ligands, and cytokines [20]. All things considered, adjuvants should be selected by their duration within the intestinal tissues and ability to activate immune pathways locally, in order to prevent systemic inflammation and potentially triggering autoimmunity.

### Modulating immune tone

One approach to circumvent the biases imposed by intestinal immune tone is to target antigen delivery to microfold (M) cells, the gateway into the lymphocyte-rich Peyer’s patches. While a host of ligands of purported M cell receptors have been tested for M cell targeting [21], these efforts have been inherently limited by an incomplete characterization of the M cell receptor repertoire. Historically, low total numbers of M cells have made them difficult to study. Emerging technologies, such as M cell differentiation in intestinal enteroids [22] and enteroid-immune cell co-culture systems [23] can help advance M cell targeting studies. Perhaps high-throughput screening of the M cell surfaceome can be utilized to identify additional M cell targets for diverse molecular cargoes, which may also vary in transcytosis capacity.

### Optimizing the route of priming

The anatomical location and dosing sequence of immunization can modulate recruitment of lymphocytes to specific regions. A classic example is the use of oral polio vaccine (OPV), which unlike the intramuscular inactivated polio vaccine (IPV), allows for the production of both mucosal and systemic immunity, effectively preventing fecal-oral viral transmission. Further, mice immunized sequentially with at least 1 dose of adjuvanted SARS-CoV-2 spike given intranasally produced specific IgG, IgA, and tissue-resident memory T cells in the upper and lower respiratory tracts, whereas intramuscular immunization alone only induced peripheral responses [24].

### Enhancing imprinting signals and lymphocyte homing

Vaccines can be administered with cofactors that help increase homing receptor imprinting on activated lymphocytes. Dendritic cells within Peyer’s patches convert vitamin A into retinoic acid, which induces expression of homing markers α4β7 and CCR9 on activated lymphocytes [25]. Various murine studies demonstrate the detrimental effects of vitamin A deficiency in mucosal lymphocyte homing [26]. Vitamin A deficiency in mice abrogated vaccine-induced mucosal homing marker expression on T lymphocytes and protection from oral challenge, however, protection was restored with oral vitamin A supplementation [26]. In humans, oral all-trans retinoic acid supplementation had similar benefits when co-administered with Vivotif vaccine (typhoid), but not with Rotarix (rotavirus) or Dukoral (cholera) [27]. More recently, the inclusion of a retinoic acid receptor agonist in a parenteral rotavirus vaccine was shown to imprint gut-specific homing receptors on lymphocytes [28]. Since B and T cells exhibit homing plasticity [25], it is possible that vaccine boosters with vitamin A supplementation can re-educate memory cells to improve mucosal responses.

### Exploiting the integrated mucosal immune system

While biased mucosal homing networks can pose an impediment for vaccine efficacy, it is also an opportunity for designing better vaccines. Since conventional parenteral immunization rarely elicits robust mucosal immunity, various studies have sought to manipulate imprinting signals to induce mucosa site-specific protection following systemic priming. These “prime and pull” strategies aim to prime systemic lymphocytes with parenteral vaccination and then recruit these cells to specific mucosal locations by application of chemokines at the sites of interest. Prime and pull has been successfully demonstrated for HSV [29], HIV [30], SARS-CoV-2 [31], and tuberculosis [32]. The Iwasaki group first showed that subcutaneous vaccination of mice with attenuated HSV-2, followed by topical vaginal application of CD8^+^ T cell-recruiting chemokines CXCL9 and CXCL10 recruited parenterally primed T cells to the reproductive tract [29]. In a similar vein to prime and pull, IPV boost after OPV prime significantly increases mucosal immunity and decreases viral shedding post-challenge, despite the fact that IPV itself does not induce mucosal immunity [33,34]. Future investigation into exploiting the integration of the mucosal system via administration of parenteral subunit vaccines or inactivated rotavirus after live oral rotavirus vaccination may offer new ways to boost mucosal immunity in endemic regions where rotavirus vaccine efficacy wanes.

Overall, it may be time to reorient our approach to mucosal vaccine design from “eliciting response” to “steering immune trafficking and shaping responses in a context-dependent manner” (**Table 1**). This will likely require both re-engineering existing vaccines to better program mucosal homing and developing new ways to orchestrate immunity across mucosal sites. Central to all of these approaches is the need to deepen understanding of the nuances of mucosal immunity to integrate antigen delivery, adjuvant selection, and microbial biases to achieve effective and durable protection.
